# L-Citrulline: Novel Pharmacotherapy to Improve Outcomes in Infants and Children at Risk of Systemic or Pulmonary Vascular Disease

**DOI:** 10.3390/ph19060896

**Published:** 2026-06-05

**Authors:** Candice D. Fike, Frederick E. Barr, Judy L. Aschner

**Affiliations:** 1 Department of Pediatrics, University of Utah Health, Salt Lake City, UT 84108, USA; 2 Diversified Search Group, St. Louis, MO 63105, USA; rick.barr@dsgco.com; 3 Center for Discovery and Innovation, Department of Pediatrics, Hackensack Meridian School of Medicine, Nutley, NJ 07110, USA; judy.aschner@hmhn.org

**Keywords:** bronchopulmonary dysplasia, pulmonary hypertension, congenital heart disease, sickle cell disease, chronic kidney disease

## Abstract

Infants and children suffering from a variety of heart, lung, and blood disorders are known to be at risk of developing systemic or pulmonary vascular disease. Despite progress made in clinical care, these patients continue to experience high morbidity and mortality. There is evidence that an impairment in the L-arginine-NO signaling pathway is involved in the pathogenesis of many of the vascular disorders afflicting children. By boosting NO production, L-citrulline, the amino acid precursor of the NO substrate L-arginine, has the potential to ameliorate vascular diseases in both the systemic and pulmonary circulations. This review will discuss the current status of the use of L-citrulline as a treatment to improve outcomes in pediatric patients suffering from disorders that place them at risk of developing systemic or pulmonary vascular disease. Future directions and the potential therapeutic use of L-citrulline in vascular diseases not yet under clinical investigation will also be discussed.

## 1. Introduction

Remarkable advances have been made in the care of a variety of heart, lung, and blood disorders in infants and children that place them at risk of developing systemic or pulmonary vascular disease. When there is sufficient consensus for care of a specific disorder, guidelines have been developed with the intent of optimizing treatment. For example, in 2015 the American Heart Association (AHA) and the American Thoracic Society (ATS) published a guideline for the diagnosis, evaluation, and treatment of pulmonary hypertension (PH) in children [1]. Unfortunately, despite following optimal pharmacotherapy as outlined in the guideline, PH progresses in some infants and children, leading to unacceptably high rates of morbidity and mortality [2]. Infants with the type of PH associated with chronic lung disease of prematurity, bronchopulmonary dysplasia (BPD), carry mortality estimates of up to 47% [3]. Children with congenital heart disease (CHD) who develop post-cardiac surgery PH have mortality rates of 22.2–54.5% [4,5]. Infants and children with several other blood and vascular disorders also suffer from alarmingly high morbidity and mortality. Children with sickle cell disease (SCD) experience excruciating episodes of pain during vaso-occlusive crises (VOC) [6]. The preceding examples highlight the need to improve the care and outcomes of pediatric patients with systemic and/or pulmonary vascular disease.

The focus of this review is to discuss L-citrulline, the amino acid precursor of the NO substrate L-arginine, as a novel therapeutic to improve outcomes for pediatric patients suffering from disorders that place them at risk of developing systemic or pulmonary vascular disease. Specifically, we will review the status of investigations evaluating the use of L-citrulline in (a) pediatric patients with congenital heart disease undergoing bypass surgery; (b) premature infants with or at high risk of developing BPD-PH; and (c) children with SCD and VOC. In addition, we will explore the potential value of L-citrulline as a therapy to prevent development of vascular diseases in understudied pediatric conditions, such as renal disease.

## 2. General Considerations for Using L-Citrulline as a Pharmacotherapy

### 2.1. Vascular Endothelial Cell NO Production

The therapeutic potential of L-citrulline is largely due to its ability to increase endogenous NO production by vascular endothelial cells. The importance of endogenous NO production in maintaining the function and integrity of both the systemic and pulmonary vascular system cannot be overstated [7,8,9]. NO regulates vascular tone by activating guanylate cyclase, leading to production of cyclic GMP, which promotes relaxation of smooth muscle cells and vasodilation. By modulating cellular proliferation, apoptosis, inflammation, and oxidative stress, NO regulates vascular structure, e.g., vascular wall remodeling, and development of new vessels, i.e., angiogenesis. In addition, by preventing platelet aggregation and fibrin formation, NO helps to regulate blood clotting and plays an essential role in preventing vascular thrombosis [10]. It is now well acknowledged that disruption of endogenous NO production contributes to the pathogenesis of a variety of vascular diseases [8,9,11,12,13,14,15]. Hence, candidates for diseases/conditions that could benefit therapeutically from L-citrulline include those that involve reduced endogenous NO production by vascular endothelial cells as part of their pathogenesis.

**Figure 1:**
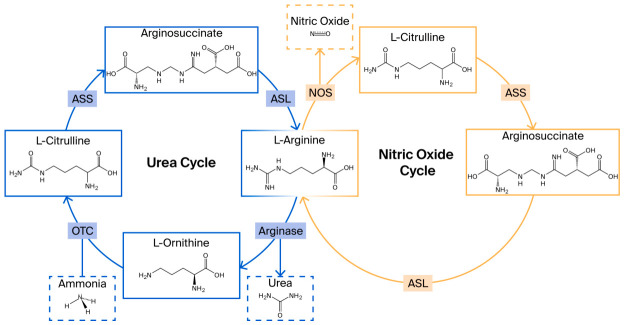
The urea cycle L-citrulline–arginine–nitric oxide metabolic pathway. NOS: Nitric oxide synthase; ASS: Argininosuccinate synthetase; ASL: Argininosuccinate lyase; OTC: Ornithine transcarbamylase.

L-citrulline drives increased NO production by a biosynthetic pathway that increases the nitric oxide synthase (NOS) substrate, L-arginine (Figure 1). This occurs via a two-step pathway involving the urea cycle enzymes, argininosuccinate synthetase (ASS) and argininosuccinate lyase (ASL) [16]. Studies evaluating the therapeutic potential of L-arginine in adult humans and animals with cardiovascular disorders have shown mixed results [17,18,19,20,21,22,23]. This is largely attributable to intestinal systemic elimination of L-arginine by arginase [23], leading to its poor bioavailability. Achievement of a significant increase in systemic L-arginine levels requires administration of very large, often poorly tolerated doses [24]. This makes L-arginine impractical for treating many patient populations, including premature infants.

In contrast, the NO-L-arginine precursor, L-citrulline, has been shown to be bioavailable when given enterally to human infants [25,26]. L-citrulline has been shown to drive endogenous NO production and inhibit the development of PH in a newborn piglet model [27,28] and in a newborn rat model [29]. When administered enterally, L-citrulline is absorbed by intestinal epithelial cells, passes through the liver without major metabolism, and reaches the systemic circulation [30,31]. In order to increase endogenous NO production, therapeutically administered L-citrulline must be transported across the vascular endothelial cell membrane [32,33,34]. Both sodium-dependent and independent plasma membrane transporters from the family of neutral amino acid transporters mediate this process [32,33,34]. Once transported into vascular endothelial cells, L-citrulline increases endogenous NO production by providing an intracellular source for the synthesis of the amino acid substrate, L-arginine, via a two-step biosynthetic pathway involving the enzymes ASS and ASL [16]. L-arginine is then synthesized to NO by endothelial NO synthase (eNOS), and during this process L-citrulline is produced. Thus, L-citrulline is part of a recycling pathway in vascular endothelial cells, serving as both a precursor for L-arginine-NO synthesis and as the end-product when L-arginine is converted to NO by eNOS [16,32,35,36,37] (Figure 1).

Of note, a portion of therapeutically administered L-citrulline may be metabolized in the kidney and reach the systemic circulation as L-arginine [38]. This is because some L-citrulline will be taken up by cells in the proximal tubules of the kidney which, like vascular endothelial cells, express ASS and ASL and can metabolize L-citrulline into L-arginine [37,39]. Hence, circulating levels of L-arginine may increase with L-citrulline administration [38]. In fact, due in part to substantial intestinal, hepatic, and systemic metabolism of L-arginine to ornithine and urea by arginase [40], enteral administration of L-citrulline, which is not a substrate of arginase, appears to be more efficient than L-arginine in raising plasma arginine levels [30,41,42,43]. Moreover, in some conditions, such as cardiopulmonary bypass or sickle cell VOC, red blood cell hemolysis occurs with the subsequent release of red blood cell arginase, causing further degradation of L-arginine. Once generated from L-citrulline, some systemically circulating L-arginine may be transported into vascular endothelial cells and be utilized by eNOS to synthesize NO.

There is substantial evidence in cell culture [32,44], tissues [27,28], and living animals [27] that administering L-citrulline is an effective way to increase NO production. Of importance, therapeutically administered L-citrulline has been shown to cause marked increases in endogenous NO production when given enterally to adult humans and children [42,45,46]. Indeed, NO production was shown to increase 3-fold in 4–16-year-old children with mitochondrial encephalomyopathy, lactic acidosis, and stroke-like episodes (MELAS) syndrome, treated enterally with L-citrulline [42]. Moreover, a greater increase in NO production occurred with enteral administration of L-citrulline than with L-arginine [42].

### 2.2. Antioxidant and Anti-Inflammatory Functions

The potential therapeutic benefit of L-citrulline extends beyond its ability to boost endogenous NO production by vascular endothelial cells. For example, independent of NO production, L-citrulline can function as an antioxidant by directly interacting with and scavenging hydroxyl radicals, which are potent reactive oxygen species (ROS) [47,48]. The ability of L-citrulline to reduce ROS has been demonstrated in vitro and is likely due to alpha-amino acids in the protonated NH_3_ state interacting with hydroxyl radicals (OH^−^), leading to formation of water and/or other unidentified products [47,48]. Other evidence of L-citrulline’s antioxidant potential is provided by studies showing that L-citrulline treatment reduced ROS production and increased amounts of the antioxidant proteins, SOD1 and SOD2, in pulmonary artery smooth muscle cells and lung tissue from a newborn rat model of lipopolysaccharide (LPS)-induced lung injury [49]. L-citrulline treatment has also been shown to reduce LPS-induced ROS generation in microvascular endothelial cells isolated from lungs of mice [50]. Certainly, some antioxidant effects of L-citrulline are related to the eNOS-NO pathway. By re-coupling eNOS in vascular endothelial cells, L-citrulline can reduce generation of the potent ROS, superoxide, and concomitantly increase NO production [32,51].

L-citrulline has also been shown to have anti-inflammatory properties via mechanisms both dependent and independent from NO production [30]. L-citrulline supplementation has been shown to reduce levels of inflammatory cytokines while not changing or increasing those of anti-inflammatory cytokines [49,52,53,54]. The anti-inflammatory function of L-citrulline involves a variety of cell types. By a process not contingent on NO generation, L-citrulline inhibited the activation of the mammalian target of rapamycin (mTOR) pathway in bone marrow-derived macrophages of adult mice and thereby suppressed production of multiple pro-inflammatory cytokines, including interleukin (IL)-6 and IL-1β [55]. In other studies, L-citrulline induced NO release and reduced generation of the pro-inflammatory cytokine, TNF-α, in peritoneal macrophages from obese diabetic rats [56]. In addition, L-citrulline pre-treatment diminished the LPS-induced generation of pro-inflammatory cytokines, IL-1β and IL-18, by microvascular endothelial cells from lungs of mice [50]. Hence, via both NO-dependent and independent mechanisms, and the involvement of cells additional to vascular endothelial cells, L-citrulline therapy could be of benefit to patients at risk of developing vascular diseases that are initiated or perpetuated by the inflammation and oxidative stress that contribute to the pathogenesis of a variety of cardiopulmonary disorders.

### 2.3. Natural Sources of L-Citrulline

L-citrulline is considered a non-essential amino acid because it can be endogenously produced by humans in the intestine and kidneys. A normal diet is a very poor source of L-citrulline in all age groups, including infants and children [57,58]. The richest known natural dietary source of L-citrulline is watermelon [57,58]. Because there is so little L-citrulline in the normal adult diet, the content of L-citrulline in human milk is negligible [30]. Moreover, L-citrulline is not currently added to either commercial formulas or parenteral nutrition solutions given to infants [35].

Rather than diet, endogenous synthesis is the main source of L-citrulline plasma levels measured in children and adults [58]. L-citrulline can be endogenously produced from ornithine and carbamoyl phosphate by mitochondrial enzymes of the urea cycle located in the liver and proximal intestine [59]. Because L-citrulline produced in the liver is compartmentalized as an intermediate of the urea cycle [31], enterocytes in the proximal intestines are the major source of circulating levels of endogenously produced L-citrulline. Enterocytes metabolize dietary L-glutamine, L-proline, and L-arginine to generate endogenous L-citrulline [31]. Therefore, dietary protein content can influence plasma L-citrulline levels. It is important to note that vascular endothelial cells do not express the enzymes needed for de novo synthesis of L-citrulline [37]. Consequently, the intracellular concentration of L-citrulline in vascular endothelial cells depends largely on uptake of blood-borne circulating L-citrulline.

The urea cycle enzymes responsible for endogenous L-citrulline production are developmentally regulated and are not expressed in the human fetus until between 13–18 weeks gestation [60,61]. The expression and activity of these enzymes increase gradually until term and do not reach mature levels until several weeks of age. Indeed, term infants have less than 50% of their adult urea cycle capacity [62]; expression of these enzymes is even lower in preterm infants. These factors contribute to the low levels of L-citrulline observed in newborn infants.

### 2.4. Therapeutic Sources of L-Citrulline

In part because of its high L-citrulline content, watermelon has been evaluated in clinical trials for its ability to improve outcomes of a variety of disorders [57,63,64,65,66,67]. Different amounts and types of watermelon product have been evaluated for therapeutic effectiveness and have included 2 cups daily of fresh diced fruit [63], 710 mL daily of watermelon puree [64], 360 mL watermelon juice twice a day [65], 4–6 g daily of watermelon extract [66,68]. A powdered form of watermelon allows for more precise control of L-citrulline doses than the fresh fruit [57]. However, the precise amounts of powder, extract, puree, juice, or fresh fruit that must be ingested to consistently achieve any specific targeted L-citrulline level and provide a beneficial effect have not yet been determined, which poses an obstacle to its therapeutic use.

Pharmaceutical/nutraceutical grade formulations of L-citrulline are commercially available for use in clinical trials and as dietary supplements. Indeed, despite inconsistent evidence of providing a beneficial effect, L-citrulline has become a popular supplement among athletes to improve performance during prolonged physical activity [69,70,71]. Some of the inconsistency in showing improved exercise performance could be due to the use of different L-citrulline formulations. For example, L-citrulline is sometimes combined with malate to further enhance exercise performance [69,71]. Malate is one of the intermediates in the tricarboxylic acid cycle (TCA cycle) and indirectly increases usable chemical energy in the form of adenosine triphosphate (ATP). However, even when the same L-citrulline formulation has been used, beneficial effects have been inconsistent [69,70,71]. All current commercially available L-citrulline formulations are in powder or pill form and must be enterally administered.

When given enterally, L-citrulline is taken up by enterocytes within the jejunum and ileum using Na^+^-dependent, neutral amino acid transporters [31,72]. L-citrulline is not catabolized by enterocytes. By bypassing significant intestinal metabolism, it is released into the portal circulation. The liver is also unable to metabolize L-citrulline. Hence, after its release from enterocytes, L-citrulline passes through the liver unmetabolized and becomes available for distribution to the whole body via the systemic circulation [31,58].

Enterally administered L-citrulline has been shown to have high bioavailability in humans. Adults who receive oral L-citrulline achieve dramatic elevations in circulating L-citrulline levels, with minimal urinary loss [73]. Plasma L-citrulline levels have also been shown to increase significantly in premature infants treated enterally with L-citrulline [25,26].

As will be discussed in sections that follow, a proprietary formulation of L-citrulline that can be administered intravenously is being used in some clinical trials. There is no current formulation of L-citrulline that can be delivered as an inhalation therapy. Therefore, until an intravenous formulation is commercially available and/or a formulation that can be administered as an inhalant is developed, the ability to tolerate an enterally administered medication places a limitation on patient populations that are good candidates for L-citrulline therapy.

## 3. Pharmacotherapy in Infants and Children with CHD Undergoing Cardiac Surgery

### 3.1. Pathophysiologic Underpinnings

Post-operative PH is a well-known, life-threatening condition suffered by children with congenital heart disease undergoing cardiac surgery [4,5,74]. It is characterized by a rapid increase in pulmonary vascular resistance (PVR) that places a sudden pressure overload on the right ventricle. The precise mechanisms underlying post-operative PH are not completely understood but involve factors that cause pulmonary vasoconstriction, endothelial dysfunction, inflammation, and hypoxia. Moreover, the post-operative acute elevation in PVR can be exacerbated by ischemia–reperfusion injury that can occur during cardio-pulmonary bypass. If not promptly recognized, a pulmonary hypertensive crisis develops, which can lead to cardiovascular collapse and death. Effective management is aimed at causing pulmonary dilation, reducing the elevation in PVR, and alleviating pressure on the right ventricle. A variety of pharmacologic agents are currently used as pulmonary vasodilators, including iNO [75]. However, since high-quality randomized trials in the pediatric population remain scarce, the use of pulmonary vasodilators is off-label and almost entirely based on clinician experience and institutional protocols. Additional key management strategies include providing adequate sedation, optimizing ventilation, and supporting systemic blood pressure and cardiac output. Unfortunately, there is a lack of consensus regarding optimal management for neonates and children following open heart surgery, and despite attempts to optimize therapy, the development of post-operative PH remains high.

### 3.2. Studies Evaluating L-Citrulline as a Potential Therapeutic Agent in Pediatric Patients with CHD Undergoing Cardiac Surgery

Pulmonary vascular endothelial function is disrupted before and during cardiac surgery [76,77,78], especially in cardiac lesions with excess pulmonary blood flow and shear stress on the pulmonary vasculature before repair. This disruption involves impaired NO bioavailability, as was demonstrated in a study showing that plasma NO metabolites were reduced in infants with CHD undergoing cardiac bypass surgery for repair of ventricular septal defects or atrioventricular septal defects [74]. These infants also experienced a perioperative reduction in plasma levels of both the eNOS-NO substrate, L-arginine, and its precursor, L-citrulline [74]. These findings provided the basis to pursue the therapeutic potential of administration of L-citrulline during the perioperative period to preserve endothelial function and inhibit the development of post-operative PH.

To evaluate L-citrulline to inhibit post-operative PH, 40 children with CHD undergoing cardiac bypass surgery at a single site were randomized to receive five doses of enterally administered L-citrulline (1.9 g/m^2^) or placebo in the perioperative period [79]. The first dose of L-citrulline or placebo was given in the operating room prior to starting cardiopulmonary bypass, and the four subsequent doses were given post-operatively in the intensive care unit over the 36 h following surgery. Notably, post-operative PH did not develop in those patients with post-operative plasma L-citrulline concentrations exceeding 37 micromolar, regardless of whether the L-citrulline level was naturally high or achieved by treatment with enteral L-citrulline [79].

Results of another small clinical trial in infants and children with CHD also supported the possibility that L-citrulline plasma levels greater than 37 micromolar might protect them from developing post-operative PH. This clinical trial, performed by a different group of investigators at a different single site, enrolled 16 children undergoing surgery for CHD and randomized them to receive five enteral doses of either placebo or L-citrulline (3 g/m^2^) [80]. The majority of L-citrulline-treated patients had post-operative plasma L-citrulline levels that exceeded 37 micromolar, and all of them had post-operative pulmonary arterial pressures that remained below 20 mmHg. By comparison, none of the placebo group had post-operative L-citrulline plasma levels exceeding 37 micromolar and 67% of them developed post-operative pulmonary arterial pressures ≥20 mmHg [80].

Of note, these two studies also provided evidence that enteral L-citrulline is safe and well-tolerated in infants and children with CHD undergoing cardiac surgery. However, a limitation of enterally administered therapies, including L-citrulline, is that variability in absorption can result in inconsistent plasma levels. Intravenous delivery can provide more predictable and consistent plasma levels but necessitates using specially prepared formulations that are proven safe and well-tolerated by the intended patient population. Prior to performing phase 3 efficacy trials, pharmacokinetic and pharmacodynamic data should be gathered to guide the choice of dosing strategies evaluated for efficacy.

With the intent to provide safety and pharmacokinetic information in pediatric patients with CHD undergoing surgery, a first-in-human study using L-citrulline specially prepared for intravenous administration was performed [81]. In the first phase of the study, eight pediatric patients received two intravenous bolus doses of either 50, 100, or 150 mg/kg L-citrulline. The first intravenous bolus dose was delivered after initiation of cardiopulmonary bypass, and the second bolus dose was given 4 h later in the pediatric critical care unit (PICU). Pharmacokinetic analysis of plasma L-citrulline levels revealed that the half-life and clearance of an intermittent bolus dosing schedule would not maintain a constant L-citrulline plasma level. Therefore, in a second phase, an additional nine patients received an intravenously administered L-citrulline bolus dose (150 mg/kg) administered after initiation of cardiopulmonary bypass, followed 4 h later in the PICU with a continuous intravenous infusion (9 mg/kg/h) of L-citrulline given for 48 h. Target L-citrulline plasma levels of 80–100 micromolar were achieved and then sustained by this dosing regimen. These higher target L-citrulline levels were intentionally chosen to be well above 37 micromolar, the plasma level that had previously been shown to provide protection from post-operative PH [79,80]. Importantly, this intravenous L-citrulline dosing regimen was found to be safe and well-tolerated by infants and children with CHD [81].

To provide evidence of potential therapeutic efficacy and confirm the safety of the intravenous dosing strategy, 22 pediatric patients, <6 years of age, undergoing cardiopulmonary bypass for surgical repair of either an atrial septal defect, ventricular septal defect, or an atrioventricular septal defect were randomized to receive either placebo or intravenously administered L-citrulline, using a modification of the second phase dosing regimen outlined above [81]. The modifications were added due to the routine introduction of modified ultrafiltration (MUF) after cardiopulmonary bypass to improve hematocrit and reduce inflammatory mediators immediately after surgery. The modifications included adding L-citrulline at a concentration of 200 micromolar to the ultrafiltration fluid and adding a post-operative L-citrulline bolus of 20 mg/kg after separation from bypass.

Results of this phase 1b study (see ClinicalTrials.gov Identifier: NCT01120964) provided preliminary evidence that intravenously administered L-citrulline might improve post-operative outcomes in patients with the type of CHD that was studied. Specifically, the study found that the duration of post-operative invasive mechanical ventilation and total duration of respiratory support, i.e., parameters that reflect the development of post-operative PH, were shorter in the L-citrulline-treated than in the placebo group. Pulmonary arterial pressures could not be used to assess post-operative PH as an efficacy outcome because of the challenges in obtaining adequate echocardiographic data. Similar numbers and types of adverse events were reported in placebo and L-citrulline-treated groups, further addressing the safety of intravenous L-citrulline in this patient population.

These promising results prompted a phase 3 RCT with the goal of determining whether L-citrulline is effective and safe in preventing the sequelae of acute lung injury in pediatric patients undergoing surgery for repair of either an atrial septal defect, ventricular septal defect, or an atrioventricular septal defect (ClinicalTrials.gov Identifier: NCT02891837). The study enrolled a total of 189 patients from 29 different centers, including 20 sites located inside the US and 9 sites located outside the US in Europe and Israel. Ninety patients were randomized to receive placebo, and 99 patients were randomized to receive intravenously administered L-citrulline, using the dosing regimen outlined in the phase 1b study. This phase 3 study found no difference between the placebo and L-citrulline-treated groups in any of the efficacy outcomes, which included length of time on post-operative mechanical ventilation and length of time on post-operative inotrope infusions. Adverse outcomes were similar in both groups. Thus, although safe to administer, IV L-citrulline was not shown to be an effective therapy to reduce sequelae of acute lung injury in pediatric patients with either an atrial septal defect, ventricular septal defect, or an atrioventricular septal defect undergoing surgery. It is notable that standard of care differed between US and non-US centers. A post hoc analysis excluding sites outside the US showed that L-citrulline significantly promoted earlier initial extubation and reduced total post-operative ventilator time and inotrope use in the US population.

The findings in the post hoc analysis of the initial Phase 3 study motivated a second Phase 3 study to be performed in centers located only in the US. The intent was to perform a large RCT in patients with a septal defect undergoing surgery in US centers and determine if intravenous L-citrulline was safe and effective in reducing the sequelae of cardiopulmonary bypass-induced acute lung injury. The goals, patient population, study protocol, and outcome measures for the second Phase 3 trial were the same as outlined for the initial Phase 3 trial (see clinicaltrials.gov. (ClinicalTrials.gov Identifier: NCT05253209). The study was terminated after 64 patients were enrolled because interim analysis revealed that it would not be feasible to enroll the number of patients required to show efficacy in any of the outcome measures.

These phase 3 studies reveal the challenges faced when designing and performing studies with the goal of providing definitive clinical evidence of therapeutic efficacy. One challenge is that it is rarely possible to standardize patient care in all study sites. It is well known that variability in routine clinical management of patients can influence outcome measurements. Another issue is that eligibility criteria used to enroll patients into a study will influence the ability to detect a change in outcome. In other words, the patient population should have a high enough risk of developing the disease, in this case post-operative PH, to be able to feasibly detect a change in outcome. It is possible that the overall risk of developing post-operative PH in patients undergoing surgery for either an atrial septal defect, ventricular septal defect, or an atrioventricular septal defect was quite variable from patient to patient and lesion to lesion, making it extremely difficult to detect an improved outcome in this patient population. The inability to provide definitive evidence of improved outcomes in the phase 3 studies performed to date does not negate the possibility that intravenous L-citrulline may be clinically effective in some pediatric patients undergoing surgery for other types of congenital heart disease.

### 3.3. Future Directions

Consideration should be given to performing studies designed to detect improved outcomes from use of intravenous L-citrulline during the perioperative period in a different patient population of infants with CHD than has been studied to date. For example, infants with single ventricle heart disease (SVHD), a particularly severe form of congenital heart disease, must undergo a series of staged operations including caval to pulmonary shunts that rely on passive pulmonary blood flow to survive into adulthood. It is critical that a low pulmonary vascular resistance be maintained in these patients during their staged operations to assure adequate pulmonary blood flow. Unfortunately, despite attempts to optimize care in the perioperative period, 27% of infants with SVHD undergoing Stage 2 palliation experience complications directly related to insufficient pulmonary blood flow, including severe hypoxemia, respiratory failure, and persistent pleural effusions [82]. Similar to the previously reported findings in infants and children with septal defects [74], it has been shown that during the 48 h post Stage 2 palliation surgery, patients with SVHD experience a reduction in plasma levels of both L-arginine and L-citrulline, the amino acids needed for NO production [83]. Moreover, SVHD patients with larger alterations in these plasma amino acid levels experienced greater post-operative morbidity [83]. These findings provide motivation for future studies designed to pursue the possibility that therapeutic administration of L-citrulline during the Stage 2 perioperative period might preserve pulmonary blood flow and improve outcomes in the SVHD patient population.

## 4. Pharmacotherapy in Infants and Children with Sickle Cell Disease (SCD) and Vaso-Occlusive Crises (VOC)

### 4.1. Pathophysiologic Underpinnings

VOCs are the most common complication of sickle cell anemia and underlie the majority of emergency room visits and hospitalizations for individuals with this disorder [6,84,85].

VOCs are the acute episodes of pain that occur when red blood cells (RBCs) become sickle-shaped and form clusters with other blood cells, including monocytes, neutrophils, and platelets [84,85,86]. These multicellular clusters adhere to the vascular endothelium, impede blood flow and oxygenation, thereby damaging blood vessels and causing infarcts of various organs, including bones and lungs [84,85,86]. Vaso-occlusion is a complex process that can be triggered by inflammation, stress, increased viscosity, decreased flow, and hemolysis [87,88]. An important factor contributing to the pathophysiology of VOC’s is that sickled RBCs are fragile and rigid, making them prone to hemolyze and release free hemoglobin into the plasma, which, in turn, decreases NO bioavailability [89]. It is worth noting that there are multiple mechanisms by which intravascular hemolysis reduces NO bioavailability. Specifically, in addition to releasing free hemoglobin, which scavenges NO by reacting with it to form methemoglobin and nitrate [90,91], intravascular hemolysis releases arginase, which hydrolyzes the NO precursor L-arginine [92]. Asymmetric dimethylarginine (ADMA), an endogenous NOS inhibitor that is abundant in RBCs, is also released during hemolysis [93]. These hemolysis-induced reductions in NO bioavailability cause vascular dysfunction, including impaired vasodilation and exaggerated vasoconstriction, and enhance the adhesion and activation of neutrophils, thereby contributing to the development of VOC’s in patients with SCD. Accordingly, treatments that restore NO bioavailability could improve vascular function and prevent the vascular complications of SCD.

### 4.2. Studies Evaluating L-Citrulline as a Potential Therapeutic Agent in Pediatric Patients with SCD

Based on its ability to enhance the arginine-NO pathway in vascular endothelial cells, L-citrulline has been evaluated for its ability to improve outcomes in children with SCD. An early pilot phase 2 clinical trial was performed in five children, ages 10–18, with the goal of providing evidence that L-citrulline could improve symptoms related to SCD [94]. All five patients were treated for 28 days with oral L-citrulline, 0.09–0.13 g/kg/d. Patient self-assessment analog scale scores were used to evaluate well-being and pain. All five patients reported an improved sense of wellness that began during the first two weeks of L-citrulline treatment. No patient experienced VOC’s during the 28 days of therapy, and no adverse effects were reported. The findings of this small pilot study support the notion that prolonged treatment with L-citrulline has the potential to prevent VOC’s and other complications suffered by patients with SCD.

Another potential use of L-citrulline in patients with SCD is as a treatment to stop VOC’s once they have started and decrease the severity and duration of pain they cause [95]. With this therapeutic use in mind, a phase 1 study was performed to generate safety information and pharmacokinetic data about the use of IV citrulline in patients with SCD [95]. In the first step of the study, four participants with SCD, who were not experiencing VOC’s, received a single IV bolus of 20 mg/kg L-citrulline. Data analysis and PK model simulation identified a dosing regimen that should achieve and maintain a target citrulline plasma concentration of 100 micromolar. In the second step of the study, while experiencing a VOC, four additional SCD participants were treated with the dosing regimen identified in the first step and found to achieve and maintain the target citrulline plasma concentrations of approximately 100 micromolar. Moreover, intravenous L-citrulline was found to be safe and well tolerated by patients participating in both study steps [95].

Based on the findings in the phase 1 study, a phase 2 study has been designed and is underway (see clinicaltrials.gov. Clinicaltrials.gov Identifier: NCT06635902). The goals of this double-blind placebo-controlled RCT are to provide evidence that intravenous L-citrulline can decrease the duration of pain in patients with SCD experiencing a VOC and to confirm that it is safe to use in this patient population. The study is randomizing patients with SCD, ages 4–21 years, experiencing a VOC, to receive high dose intravenous L-citrulline (50 mg/kg IV bolus followed by 9 mg/kg/h continuous infusion for 16 h), low dose intravenous L-citrulline (25 mg/kg IV bolus followed by 9 mg/kg/h continuous infusion for 16 h), or placebo. The participants are monitored for any adverse events, and the primary outcome is time-to-crises resolution. All participants receive the usual standard of care treatment for VOC’s, which may include opioids.

### 4.3. Future Directions

Pending results of the above-described phase 2 study, a subsequent large-scale multi-center phase 3 is being designed to evaluate the effectiveness of administering IV L-citrulline as a treatment to reduce the severity and duration of pain and reduce use of opioid pain medications experienced by patients with SCD during VOC’s. Consideration should also be given to performing studies designed to pursue the potential for prolonged treatment with oral L-citrulline to prevent VOC’s and other complications suffered by patients with SCD. These latter studies could include evaluating the ability of chronic oral L-citrulline treatment to improve long-term outcomes, such as preventing the development of pulmonary hypertension in patients with SCD [96].

## 5. Pharmacotherapy in Premature Infants at Risk of BPD-PH

### 5.1. Pathophysiologic Underpinnings of BPD-PH

**Figure 2:**
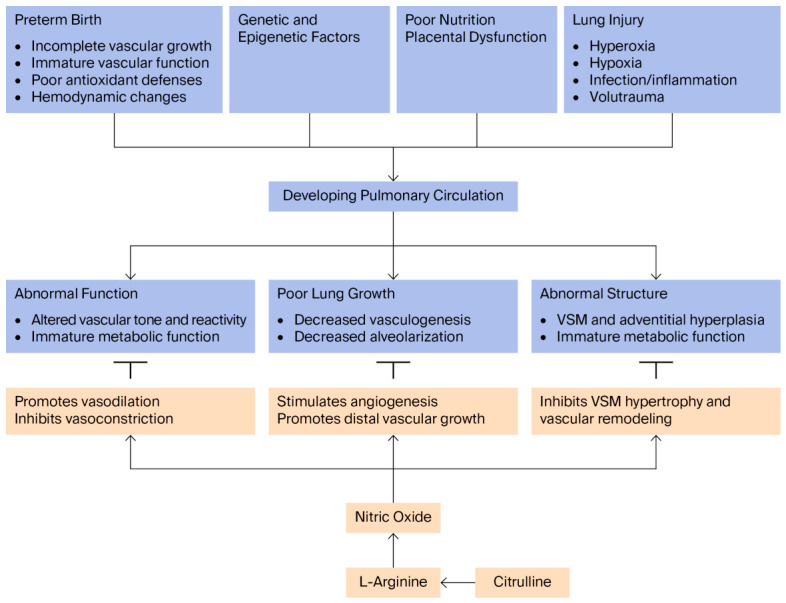
Beneficial effects of L-citrulline on the developing pulmonary circulation.

BPD is the most common form of chronic lung disease and pulmonary morbidity in premature infants. Between 8–42% of premature infants with BPD develop PH [97,98,99,100,101,102,103,104]. The survival rates for infants with BPD-PH have not improved since the 1980s and remain alarmingly low (only 50–60% survival) [103,104,105]. For decades, the standard of care for management of BPD-PH has been to attempt to treat the underlying lung disorder and to judiciously use oxygen as a pulmonary vasodilator [106,107]. No pharmacological therapy has been rigorously evaluated for efficacy for infants with BPD-PH. Nonetheless, a variety of agents, including inhaled NO and sildenafil, are currently used off-label as pulmonary vasodilators in this patient population [1,108,109,110,111,112,113]. Of importance, in addition to being a potent pulmonary vasodilator, NO promotes angiogenesis and alveolarization [114] and inhibits pulmonary vascular wall smooth muscle growth [115,116]. These multiple effects of NO on lung structure and function are important because the therapeutic goals for BPD-PH should not be limited to pulmonary vasodilation. When premature infants are exposed to conditions injurious to the lung, including exposure to positive pressure respiratory support and oxygen, the alveoli and pulmonary vasculature fail to develop normally, both structurally and functionally [117,118,119,120]. The structural abnormalities include pulmonary vascular wall thickening and failure to develop the distal pulmonary circulation [117,118,119,120]. Functional impairments include a reduced ability to vasodilate and exaggerated vasoconstrictor responses [117,118,119,120]. Therapies that improve NO production are therefore ideal candidates to evaluate for efficacy to treat BPD-PH because of the potential of NO to inhibit the many abnormalities in pulmonary vascular function and structure that are known to contribute to the pathogenesis of this devastating disease (Figure 2).

### 5.2. Studies Evaluating L-Citrulline as a Potential Therapeutic Agent in Premature Infants with or at Risk of BPD-PH

Proof of concept that improving NO bioavailability with L-citrulline could be an efficacious PH therapy was provided from pre-clinical studies with a newborn piglet model of hypoxic pulmonary hypertension. In addition to increasing pulmonary vascular NO production, enteral treatment with L-citrulline inhibited both the onset and progression of PH in piglets exposed to chronic hypoxia [27,28]. An additional pre-clinical study performed in a newborn rat model of BPD showed that pathologic evidence of PH, including right ventricular hypertrophy and pulmonary vascular remodeling, was ameliorated by treatment with L-citrulline [29]. In premature infants with BPD, those who did not develop PH had median plasma concentrations of 36 micromolar, whereas lower median plasma L-citrulline concentrations were found in those infants with BPD who developed PH [121]. Findings from these studies provide motivation to determine whether L-citrulline treatment might improve outcomes in premature infants at risk of developing BPD-PH.

In accordance with current FDA guidance, early-phase studies must first establish safety, pharmacokinetics, and pharmacodynamics in the relevant patient population before initiating a phase 3 efficacy trial. Therefore, as a first step, a phase 0 study was performed to generate pharmacokinetic and safety data about enterally administered L-citrulline in premature infants at risk of developing BPD-PH [25]. The first stage of the study enrolled 10 premature infants at high risk for developing BPD-PH. Each participant was given a single enterally administered dose of 150 mg/kg L-citrulline, which was tolerated without adverse effects. Using data derived from these 10 infants [25], a population PK (popPK) model was generated and used to determine a multi-dose regimen of L-citrulline that was evaluated for tolerance when given to premature infants enrolled in a 2nd stage of the study [122]. The multi-dose regimen of L-citrulline used in stage 2 was chosen with the goal of achieving steady-state L-citrulline concentrations of 50–80 micromolar, i.e., well above the 37 micromolar L-citrulline concentration that has previously been associated with protecting pediatric patients [79], including premature infants [121], from developing PH. All six participants enrolled in stage 2 tolerated an enterally administered multi-dose L-citrulline regimen of 60 mg/kg given four times a day (240 mg/kg/d) for 72 h, without serious adverse effects, but only two of the participants achieved the target L-citrulline concentration [122]. Taken altogether, findings from the 2 stages of the study delineate a PK profile and provide evidence that enteral L-citrulline is safe to use in premature infants at risk of developing BPD-PH [25,122].

In addition to preventing BPD-PH, it is possible that L-citrulline could be an effective treatment for BPD-PH once it is established. As a first step in pursuit of this possibility, an early phase clinical trial has been designed and is enrolling patients (see clinicaltrials.gov (ClinicalTrials.gov Identifier: NCT05636397). The goal of the study is to provide safety and PK data about the use of one of two enterally administered L-citrulline dosing regimens (either 300 or 500 mg/kg/d divided q 6 h) in premature infants with established BPD (with or without PH).

### 5.3. Future Directions

Now that PK and safety data are available for using enteral L-citrulline in premature infants at high risk of developing BPD-PH [25,122], the next step needed is to design an early-stage, dose escalation study to provide pharmacodynamic information and confirm safety of enteral L-citrulline in this premature patient population. Ideally, such a study will incorporate endpoints to reflect potential efficacy. Findings from this next-step study are needed to inform the optimal dosing regimen of L-citrulline to be evaluated for efficacy in a subsequent phase 3 study. Moreover, once the early phase study in patients with established BPD-PH is completed, and if the enteral L-citrulline is shown to be safe, the study findings can be used to design studies of L-citrulline to treat established BPD-PH.

Consideration should also be given to evaluate the potential for L-citrulline to ameliorate PH associated with other chronic lung conditions in children. Due to the need for therapies to promote lung parenchymal and pulmonary vascular growth, L-citrulline could be considered as a treatment in infants with congenital diaphragmatic hernia, a patient population that is born with hypoplastic lungs and is at high risk to developing PH.

## 6. Pharmacotherapy for Pediatric Patients with Renal Disease

**Figure 3:**
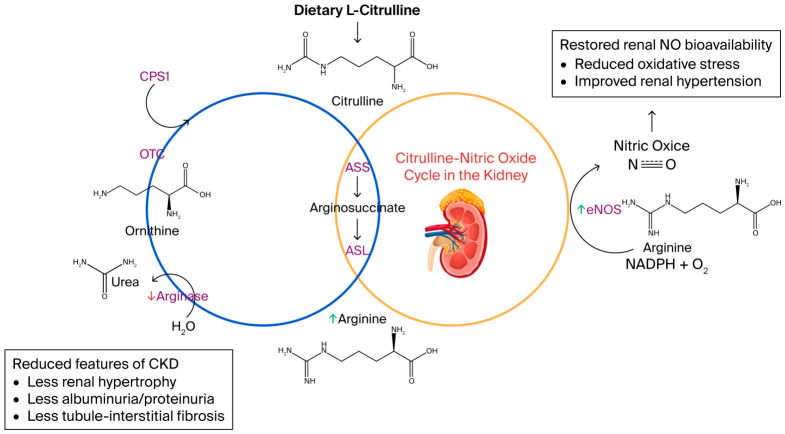
Proposed benefits of L-citrulline in renal disease. eNOS: endothelial nitric oxide synthase; NADPH: nicotine adenine dinucleotide phosphate; ASS: Argininosuccinate synthetase; ASL: Argininosuccinate lyase; OTC: Ornithine transcarbamylase, CPS1: carbamoyl phosphate synthetase 1, red downward arrow: inhibit Arginase; green upward arrows: increase Arginine amounts and promote Nitric Oxide produced from eNOS.

There is a well-recognized and bidirectional relationship between systemic hypertension and renal disease. In children, chronic kidney disease (CKD) causes secondary hypertension, which can accelerate progression of renal damage. Hypertension as a consequence of pre-existing renal disease or impairment of renal development, as can occur in extremely preterm infants, differs from adult kidney disease, where more often hypertension causes the renal failure. There is a mechanistic rationale for the use of L-citrulline for children and adults with renal disease, although definitive studies in humans are lacking. The therapeutic rationale relates to the ability of L-citrulline to bypass hepatic first-pass metabolism and be converted to L-arginine to promote endogenous NO production from eNOS (Figure 3). There is also some evidence that L-citrulline can inhibit arginase, which competes with eNOS for L-arginine. This suggests that therapeutic L-citrulline may be relevant in kidney disease where endothelial dysfunction, NO deficiency and oxidative stress contribute to disease onset and progression [54].

Evidence for L-citrulline for renal disease is derived from several adult and juvenile animal models. In young spontaneously hypertensive rats, L-citrulline therapy prevented the development of hypertension by restoring renal NO bioavailability and reducing oxidative stress [123]. In a mouse model of streptozotocin-induced type 1 diabetes, treatment with L-citrulline significantly reduced renal hypertrophy, albuminuria, and tubule-interstitial fibrosis. An anti-inflammatory profile was also seen with L-citrulline supplementation, with increased IL-10 levels reported in the L-citrulline-treated mice [54]. In a model of diabetes-induced renal disease in adult rats, L-citrulline, but not L-arginine, ameliorated glomerular hyperfiltration and proteinuria [124]. However, there is conflicting evidence in other animal models. In another study in diabetic mice, although L-citrulline markedly increased L-arginine levels in plasma and in the kidney, neither oral L-citrulline nor oral L-arginine prevented or reduced albuminuria, elevations in blood urea nitrogen, or histopathological changes in the glomeruli [125]. The inconsistent findings in these various animal models may relate to differences in disease stage at the time of supplementation. It may also relate to differences in endothelial dysfunction across kidney disease etiologies. A better understanding of the underlying mechanism of renal disease may help predict L-citrulline responsiveness and explain why treatment with L-citrulline may work in some disease states but not others.

In CKD, it has been established that renal arginine synthesis is impaired because of loss of ASS and ASL early in disease development [126], which raises the possibility that L-citrulline supplementation can compensate for enzymatic deficiencies and reverse features of CKD. In a mouse model, it was demonstrated that extrarenal tissues can use citrulline to produce arginine when kidney function is impaired, although with less efficiency than a normal functioning kidney [127].

Unfortunately, data from human clinical trials are currently unavailable. A review from 2018 noted that L-citrulline shows promise in a variety of cardiovascular diseases and in diseases caused by malnutrition, but there is a need for formal clinical trials in human kidney disease [128]. A 2024 review also emphasized the need for research on optimal L-citrulline dosing and efficacy in renal hypertension and other forms of cardiovascular diseases where NO deficiency and endothelial dysfunction play a role in pathogenesis [129].

## 7. Summary and Conclusions

L-citrulline is a natural substance that is being evaluated as a potential treatment for the vascular complications that accompany a variety of childhood disorders, such as post-operative PH in children with CHD, VOC’s in children with SCD, and PH associated with BPD. Evidence has been provided showing that an impairment in the L-arginine NO signaling pathway is involved in the pathogenesis of each of these pediatric disorders. It follows logically that the ability to boost NO production is a major reason that L-citrulline, the amino acid precursor of the NO substrate, L-arginine, holds promise to improve outcomes in these patients. In accordance with FDA guidance, prior to performing phase 3 efficacy trials, early phase studies have been performed or are underway to delineate the pharmacokinetics of L-citrulline in pediatric patients with each of these disease conditions. Importantly, L-citrulline has been found to be safe and well tolerated in all pediatric patient populations studied to date. Definitive evidence that L-citrulline is an effective treatment for any specific pediatric vascular disease awaits results from future carefully designed and rigorously performed phase 3 RCTs. Animal models provide pre-clinical data that supports performing clinical trials of L-citrulline as a treatment for systemic hypertension associated with renal disease. Moreover, L-citrulline merits consideration as a therapeutic option for vascular diseases, not yet under investigation, that involve dysregulated NO biology in the pediatric and adult populations.

## Data Availability

No new data were created or analyzed in this study. Data sharing is not applicable.

## References

[1] Abman S.H., Hansmann G., Archer S.L., Ivy D.D., Adatia I., Chung W.K., Hanna B.D., Rosenzweig E.B., Raj J.U., Cornfield D. (2015). Pediatric Pulmonary Hypertension: Guidelines from the American Heart Association and American Thoracic Society. Circulation.

[2] Hayes D., Jennerich A.L., Coleman R.D., Abston E., Adamson G.T., Berger J.T., Cohen S.P., Cooper D.S., Eghtesady P., Fynn-Thompson F. (2025). Interventional Strategies for Children with Progressive Pulmonary Hypertension Despite Optimal Therapy: An Official American Thoracic Society Clinical Practice Guideline. Am. J. Respir. Crit. Care Med..

[3] Hansmann G., Sallmon H., Roehr C.C., Kourembanas S., Austin E.D., Koestenberger M., European Pediatric Pulmonary Vascular Disease Network (2021). Pulmonary hypertension in bronchopulmonary dysplasia. Pediatr. Res..

[4] Hopkins R.A., Bull C., Haworth S.G., de Leval M.R., Stark J. (1991). Pulmonary hypertensive crises following surgery for congenital heart defects in young children. Eur. J. Cardiothorac. Surg..

[5] Lindberg L., Olsson A.K., Jogi P., Jonmarker C. (2002). How common is severe pulmonary hypertension after pediatric cardiac surgery?. J. Thorac. Cardiovasc. Surg..

[6] Lanzkron S., Carroll C.P., Haywood C. (2010). The burden of emergency department use for sickle-cell disease: An analysis of the national emergency department sample database. Am. J. Hematol..

[7] Ghimire K., Altmann H.M., Straub A.C., Isenberg J.S. (2017). Nitric oxide: What’s new to NO?. Am. J. Physiol. Cell Physiol..

[8] Gonzalez M., Clayton S., Wauson E., Christian D., Tran Q.K. (2025). Promotion of nitric oxide production: Mechanisms, strategies, and possibilities. Front. Physiol..

[9] Tejero J., Shiva S., Gladwin M.T. (2019). Sources of Vascular Nitric Oxide and Reactive Oxygen Species and Their Regulation. Physiol. Rev..

[10] Loscalzo J. (2001). Nitric oxide insufficiency, platelet activation, and arterial thrombosis. Circ. Res..

[11] Benza R.L., Grunig E., Sandner P., Stasch J.P., Simonneau G. (2024). The nitric oxide-soluble guanylate cyclase-cGMP pathway in pulmonary hypertension: From PDE5 to soluble guanylate cyclase. Eur. Respir. Rev..

[12] D’Agostino A., Lanzafame L.G., Buono L., Crisci G., D’Assante R., Leone I., De Vito L., Bossone E., Cittadini A., Marra A.M. (2023). Modulating NO-GC Pathway in Pulmonary Arterial Hypertension. Int. J. Mol. Sci..

[13] Klinger J.R., Abman S.H., Gladwin M.T. (2013). Nitric oxide deficiency and endothelial dysfunction in pulmonary arterial hypertension. Am. J. Respir. Crit. Care Med..

[14] Tonelli A.R., Haserodt S., Aytekin M., Dweik R.A. (2013). Nitric oxide deficiency in pulmonary hypertension: Pathobiology and implications for therapy. Pulm. Circ..

[15] Tettey A., Jiang Y., Li X., Li Y. (2021). Therapy for Pulmonary Arterial Hypertension: Glance on Nitric Oxide Pathway. Front. Pharmacol..

[16] Solomonson L.P., Flam B.R., Pendleton L.C., Goodwin B.L., Eichler D.C. (2003). The caveolar nitric oxide synthase/arginine regneration system for NO production in endothelial cells. J. Exp. Biol..

[17] Baudouin S.V., Bath P., Martin J.F., Bois R.D., Evans T.W. (1993). L-arginine infusion has no effect on systemic haemodynamics in normal volunteers, or lsytemic and pulmonary hemodynamics in patients with elevated pulmonary vascular resistance. Br. J. Clin. Pharmac..

[18] Brown M.B., Kempf A., Collins C.M., Long G.M., Owens M., Gupta S., Hellman Y., Wong V., Farber M., Lahm T. (2018). A prescribed walking regimen plus arginine supplementation improves function and quality of life for patients with pulmonary arterial hypertension: A pilot study. Pulm. Circ..

[19] Laursen B.E., Dam M.Y., Mulvany M.J., Simonsen U. (2008). Hypoxia-induced pulmoanry vascular remodeling and right ventricular hypertrophy is unaltered by long-term oral L-arginine administration. Vasc. Pharmacol..

[20] Nagaya N., Uematshu M., Oya H., Sato N., Sakamaki F., Kyotani S., Ueno K., Nakanishi N., Yamagishi M., Miyatake K. (2001). Short-term oral administration of L-arginine improves hemodynamics and excercise capacity in patientw with precapillary pulmonary hypertension. Am. J. Respir. Crit. Care Med..

[21] Mitani Y., Maruyama K., Sakurai M. (1997). Prolonged administration of L-arginine ameliorates chronic pulmonary hypertension and pulmonary vascular remodeling in rats. Circulation.

[22] Ou Z.-J., Wei W., Huang D., Luo D., Wang Z., Zhang X., Ou J. (2010). L-arginine restores endothelial nitric oxide synthase coupled activity and attenuates monocrotaline-induced pulmonary artery hypertension in rats. Am. J. Physiol. Endocrinol. Metab..

[23] Schulman S.P., Becker L.C., Kass D.A., Champion H.C., Terrin M.L., Forman S., Ernst K.V., Kelemen M.D., Townsend S.N., Capriotti A. (2006). L-arginine therapy in acute myocardial infarction: The vascular interaction with age in myocardial infarction (VINTAGE MI) randomized clinical trial. JAMA.

[24] Tenenbaum A., Fisman E.Z., Motro M. (1998). L-arginine: Rediscovery in progress. Cardiology.

[25] Fike C.D., Avachat C., Birnbaum A.K., Aschner J.L., Sherwin C.M. (2023). Pharmacokinetics of L-Citrulline in Neonates at Risk of Developing Bronchopulmonary Dysplasia-Associated Pulmonary Hypertension. Paediatr. Drugs.

[26] Qasim A., Mehdi M.Q., Bhatia S., Franco-Fuenmayor M.E., Jain S.K. (2024). Enteral L-citrulline supplementation in preterm infants is safe and effective in increasing plasma arginine and citrulline levels-a pilot randomized trial. J. Perinatol..

[27] Ananthakrishnan M., Barr F.E., Summar M.L., Smith H.A., Kaplowitz M., Cunningham G., Magarik J., Zhang Y., Fike C.D. (2009). L-Citrulline ameliorates chronic hypoxia-induced pulmonary hypertension in newborn piglets. Am. J. Physiol. Lung Cell. Mol. Physiol..

[28] Fike C.D., Dikalova A., Kaplowitz M.R., Cunningham G., Summar M., Aschner J.L. (2015). Rescue Treatment with L-Citrulline Inhibits Hypoxia-Induced Pulmonary Hypertension in Newborn Pigs. Am. J. Respir. Cell Mol. Biol..

[29] Vadivel A., Aschner J.L., Rey-Parra G.J., Magarik J., Zeng H., Summar M., Eaton F., Thebaud B. (2010). L-citrulline attenuates arrested alveolar growth and pulmonary hypertension in oxygen-induced lung injury in newborn rats. Pediatr. Res..

[30] Allerton T.D., Proctor D.N., Stephens J.M., Dugas T.R., Spielmann G., Irving B.A. (2018). L-citrulline supplementation: Impact on cardiometabolic health. Nutrients.

[31] Curis E., Nicolis I., Moinard C., Osowska S., Zerrouk N., Benazeth S., Cynober L. (2005). Almost all about citrulline in mammals. Amino Acids.

[32] Dikalova A., Fagiana A., Aschner J.L., Aschner M., Summar M., Fike C.D. (2014). Sodium-coupled neutral amino acid transporter 1 (SNAT 1) modulates L-citrulline transport and nitric oxide (NO) signaling in piglet pulmonary arterial endothelial cells. PLoS ONE.

[33] Fike C.D., Sidoryk-Wegrzynowicz M., Aschner M., Summar M., Prince L.S., Cunningham G., Kaplowitz M., Zhang Y., Aschner J.L. (2012). Prolonged hypoxia augments L-citrulline transport by System A in the newborn piglet pulmonary circulation. Cardiovasc. Res..

[34] Mackenzie B., Erickson J.D. (2004). Sodium-coupled neutral amino acid (System N/A) transporters of the SLC38 gene family. Pflug. Arch. Eur. J. Physiol..

[35] Mascarenhas D., Mohammadi A., Higazy R., Ivanovska J., Gauda E., Jasani B. (2024). L-Citrulline in Neonates: From Bench to Bed Side. Children.

[36] Erez A., Nagamani S.C., Shchelochkov O.A., Premkumar M.H., Campeau P.M., Chen Y., Garg H.K., Li L., Mian A., Bertin T.K. (2011). Requirement of argininosuccinate lyase for systemic nitric oxide production. Nat. Med..

[37] Neill M.A., Aschner J., Barr F., Summar M.L. (2009). Quantitative RT-PCR comparison of the urea and nitric oxide cycle gene transcripts in adult human tissues. Mol. Genet. Metab..

[38] Castillo L., Chapman T.E., Sanchez M., Yu Y.M., Burke J.F., Ajami A.M., Vogt J., Young V.R. (1993). Plasma arginine and citrulline kinetics in adults given adequate and arginine-free diets. Proc. Natl. Acad. Sci. USA.

[39] Levillain O., Parvy P., Hassler C. (1997). Amino acid handling in uremic rats: Citrulline, a reliable marker of renal insufficiency and proximal tubular dysfunction. Metabolism.

[40] Morris S.M. (2002). Regulation of enzymes of the urea cycle and arginine metabolism. Annu. Rev. Nutr..

[41] Bahadoran Z., Mirmiran P., Kashfi K., Ghasemi A. (2021). Endogenous flux of nitric oxide: Citrulline is preferred to Arginine. Acta Physiol..

[42] El-Hattab A.W., Emrick L.T., Hsu J.W., Chanprasert S., Almannai M., Craigen W.J., Jahoor F., Scaglia F. (2016). Impaired nitric oxide production in children with MELAS syndrome and the effect of arginine and citrulline supplementation. Mol. Genet. Metab..

[43] Mori M., Gotoh T. (2000). Regulation of nitric oxide production by arginine metabolic enzymes. Biochem. Biophys. Res. Commun..

[44] Dikalova A., Aschner J.L., Zhang Y., Kaplowitz M.R., Fike C.D. (2019). Reactive oxygen species modulate Na^+^-coupled neutral amino acid transporter 1 expression in piglet pulmonary artery endothelial cells. Am. J. Physiol. Heart Circ. Physiol..

[45] El-Hattab A.W., Hsu J.W., Emrick L.T., Wong L.J., Craigen W.J., Jahoor F., Scaglia F. (2012). Restoration of impaired nitric oxide production in MELAS syndrome with citrulline and arginine supplementation. Mol. Genet. Metab..

[46] Schwedhelm E., Maas R., Freese R., Jung D., Lukacs Z., Jambrecina A., Spickler W., Schulze F., Boger R.H. (2008). Pharmacokinetic and pharmacodynamic properties of oral L-citrulline and L-arginine: Impact on nitric oxide metabolism. Br. J. Clin. Pharmacol..

[47] Akashi K., Miyake C., Yokota A. (2001). Citrulline, a novel compatible solute in drought-tolerant wild watermelon leaves, is an effecient hydroxyl radical scavenger. FEBS Lett..

[48] Nagy I., Floyd R.A. (1984). Hydroxyl free radical reactions with amino acids and proteins studied by electron spin resonance spectroscopy and spin-trapping. Biochim. Biophys. Acta.

[49] Ivanovski N., Wang H., Tran H., Ivanovska J., Pan J., Miraglia E., Leung S., Posiewko M., Li D., Mohammadi A. (2023). L-citrulline attenuates lipopolysaccharide-induced inflammatory lung injury in neonatal rats. Pediatr. Res..

[50] Xue Y., Zhang Y., Chen L., Wang Y., Lv Z., Yang L.Q., Li S. (2022). Citrulline protects against LPS-induced acute lung injury by inhibiting ROS/NLRP3-dependent pyroptosis and apoptosis via the Nrf2 signaling pathway. Exp. Ther. Med..

[51] Douglass M.S., Kaplowitz M.R., Zhang Y., Fike C.D. (2023). Impact of l-citrulline on nitric oxide signaling and arginase ativity in hypoxic human pulmonary artery endothelial cells. Pulm. Circ..

[52] Asgeirsson T., Zhang S., Nunoo R., Mascarenas C., Dujovny N., Luchtefeld M., Cavey G.S., Senagore A. (2011). Citrulline: A potential immunomodulator in sepsis. Surgery.

[53] Ho S.W., El-Nezami H., Corke H., Ho C.S., Shah N.P. (2022). L-citrulline enriched fermented milk with Lactobacillus helveticus attenuates dextran sulfate sodium (DSS) induced colitis in mice. J. Nutr. Biochem..

[54] Romero M.J., Yao L., Sridhar S., Bhatta A., Dou H., Ramesh G., Brands M.W., Pollock D.M., Caldwell R.B., Cederbaum S.D. (2013). l-Citrulline Protects from Kidney Damage in Type 1 Diabetic Mice. Front. Immunol..

[55] Xie Z., Lin M., Xing B., Wang H., Zhang H., Cai Z., Mei X., Zhu Z.J. (2025). Citrulline regulates macrophage metabolism and inflammation to counter aging in mice. Sci. Adv..

[56] Breuillard C., Bonhomme S., Couderc R., Cynober L., De Bandt J.P. (2015). In vitro anti-inflammatory effects of citrulline on peritoneal macrophages in Zucker diabetic fatty rats. Br. J. Nutr..

[57] Baiao D.D.S., da Silva D.V.T., Paschoalin V.M.F. (2025). Watermelon Nutritional Composition with a Focus on L-Citrulline and Its Cardioprotective Health Effects-A Narrative Review. Nutrients.

[58] Windmueller H.G., Spaeth A.E. (1981). Source and fate of circulating citrulline. Am. J. Physiol. Endocrinol. Metab..

[59] Morris S.M. (2009). Recent advances in arginine metabolism: Roles and regulation of the arginases. Br. J. Pharmacol..

[60] Kohler E.S., Sankaranarayanan S., van Ginneken C.J., van Dijk P., Vermeulen J.L., Ruijter J.M., Lamers W.H., Bruder E. (2008). The human neonatal small intestine has the potential for arginine synthesis; developmental changes in the expression of arginine-synthesizing and -catabolizing enzymes. BMC Dev. Biol..

[61] Mukarram Ali Baig M., Habibullah C.M., Swamy M., Hassan I., Taher U.Z., Ayesha Q., Devi B.G. (1992). Studies on urea cycle enzyme levels in the human fetal liver at different gestational ages. Pediatr. Res..

[62] Pearson D.L., Dawling S., Walsh W.F., Haines J.L., Christman B.W., Bazyk A., Scott N., Summar M.L. (2001). Neonatal pulmonary hypertension: Urea-cycle intermediates, nitric oxide production, and carbamoyl-phosphate synthetase function. N. Engl. J. Med..

[63] Ochiai M., Hayashi T., Morita M., Ina K., Maeda M., Watanabe F., Morishita K. (2012). Short-term effects of L-citrulline supplementation on arterial stiffness in middle aged-men. Int. J. Cardiol..

[64] Shanely R.A., Zwetsloot J.J., Jurrissen T.J., Hannan L.C., Zwetsloot K.A., Needle A.R., Bishop A.E., Wu G., Perkins-Veazie P. (2020). Daily watermelon consumption decreases plasma sVCAM-1 levels in overweight and obese postmenopausal women. Nutr. Res..

[65] Ellis A.C., Mehta T., Nagabooshanam V.A., Dudenbostel T., Locher J.L., Crowe-White K.M. (2021). Daily 100% watermelon juice consumption and vascular function among postmenopausal women: A randomized controlled trial. Nutr. Metab. Cardiovasc. Dis..

[66] Figueroa A., Wong A., Hooshmand S., Sanchez-Gonzalez M.A. (2013). Effects of watermelon supplementation on arterial stiffness and wave reflection amplitude in postmenopausal women. Menopause.

[67] Volino-Souza M., Oliveira G.V., Conte-Junior C.A., Figueroa A., Alvares T.S. (2022). Current Evidence of Watermelon (Citrullus lanatus) Ingestion on Vascular Health: A Food Science and Technology Perspective. Nutrients.

[68] Figueroa A., Sanchez-Gonzalez M.A., Wong A., Arjmandi B.H. (2012). Watermelon extract supplementation reduces ankle blood pressure and carotid augmentation index in obese patients with prehypertension or hypertension. Am. J. Hypertens..

[69] Duttagupta S., Krishna Roy N., Dey G. (2024). Efficacy of amino acids in sports nutrition- review of clinical evidences. Food Res. Int..

[70] Sureda A., Pons A. (2012). Arginine and citrulline supplementation in sports and exercise: Ergogenic nutrients?. Med. Sport Sci..

[71] Trexler E.T., Persky A.M., Ryan E.D., Schwartz T.A., Stoner L., Smith-Ryan A.E. (2019). Acute effects of citrulline supplementation on high-intensity strength and poser performance: A systematic review and meta-analysis. Sports Med..

[72] Vadgama J.V., Evered D.F. (1992). Characteristics of L-citrulline transport across rat small intestine in vitro. Ped. Res..

[73] Rouge C., Robert C.D., Robbins A., Bacquer O.L., Volteau C., Cochetiere M.D.L., Darmaun D. (2007). Manipulation of citrulline availability in humans. Am. J. Physiol. Gastrointest. Liver Physiol..

[74] Barr F.E., Beverley H., VanHook K., Cermak E., Christian K., Drinkwater D., Dyer K., Raggio N.T., Moore J.H., Christman B. (2003). Effect of cardiopulmonary bypass on urea cycle intermediates and nitric oxide levels after congenital heart surgery. J. Pediatr..

[75] Curran R.D., Mavroudis C., Backer C.L., Sautel M., Zales V.R., Wessel D.L. (1995). Inhaled nitric oxide for children with congenital heart disease and pulmonary hypertension. Ann. Thorac. Surg..

[76] Steinhorn R.H., Fineman J.R. (1999). The pathophysiology of pulmonary hypertension in congenital heart disease. Artif. Organs.

[77] Schulze-Neick I., Li J., Penny D.J., Redington A.N. (2001). Pulmonary vascular resistance after cardiopulmonary bypass in infants: Effect on postoperative recovery. J. Thorac. Cardiovasc. Surg..

[78] Kirshbom P.M., Jacobs M.T., Tsui S.S., DiBernardo L.R., Schwinn D.A., Ungerleider R.M., Gaynor J.W. (1996). Effects of cardiopulmonary bypass and circulatory arrest on endothelium-dependent vasodilation in the lung. J. Thorac. Cardiovasc. Surg..

[79] Smith H.A., Canter J.A., Christian K.G., Drinkwater D.C., Scholl F.G., Christman B.W., Rice G.D., Barr F.E., Summar M.L. (2006). Nitric oxide precursors and congenital heart surgery: A randomized controlled trial of oral citrulline. J. Thorac. Cardiovasc. Surg..

[80] Silvera Ruiz S., Grosso C.L., Tablada M., Cabrera M., Dodelson de Kremer R., Juaneda E., Larovere L.E. (2020). Efficacy of citrulline supplementation to decrease the risk of pulmonary hypertension after congenital heart disease surgery. A local experience. Rev. Fac. Cienc. Med. Cordoba.

[81] Barr F.E., Tirona R.G., Taylor M.B., Rice G., Arnold J., Cunningham G., Smith H.A., Campbell A., Canter J.A., Christian K.G. (2007). Pharmacokinetics and safety of intravenously administered citrulline in children undergoing congenital heart surgery: Potential therapy for postoperative pulmonary hypertension. J. Thorac. Cardiovasc. Surg..

[82] Kogon B.E., Plattner C., Leong T., Simsic J., Kirshbom P.M., Kanter K.R. (2008). The bidirectional Glenn operation: A risk factor analysis for morbidity and mortality. J. Thorac. Cardiovasc. Surg..

[83] Frank B.S., Niemiec S., Khailova L., Mancuso C.A., Lehmann T., Mitchell M.B., Morgan G.J., Twite M., DiMaria M.V., Klawitter J. (2024). Arginine-NO metabolites are associated with morbidity in single ventricle infants undergoing stage 2 palliation. Pediatr. Res..

[84] Zaidi A.U., Glaros A.K., Lee S., Wang T., Bhojwani R., Morris E., Donohue B., Paulose J., Iorga S.R., Nellesen D. (2021). A systematic literature review of frequency of vaso-occlusive crises in sickle cell disease. Orphanet J. Rare Dis..

[85] Ballas S.K., Gupta K., Adams-Graves P. (2012). Sickle cell pain: A critical reappraisal. Blood.

[86] Darbari D.S., Sheehan V.A., Ballas S.K. (2020). The vaso-occlusive pain crisis in sickle cell disease: Definition, pathophysiology, and management. Eur. J. Haematol..

[87] Platt O.S. (2000). Sickle cell anemia as an inflammatory disease. J. Clin. Investig..

[88] Connes P., Renoux C., Joly P., Nader E. (2023). Vascular pathophysiology of sickle cell disease. Presse Med..

[89] Nader E., Conran N., Romana M., Connes P. (2021). Vasculopathy in Sickle Cell Disease: From Red Blood Cell Sickling to Vascular Dysfunction. Compr. Physiol..

[90] Reiter C.D., Wang X., Tanus-Santos J.E., Hogg N., Cannon R.O., Schechter A.N., Gladwin M.T. (2002). Cell-free hemoglobin limits nitric oxide bioavailability in sickle-cell disease. Nat. Med..

[91] Kato G.J., Gladwin M.T., Steinberg M.H. (2007). Deconstructing sickle cell disease: Reappraisal of the role of hemolysis in the development of clinical subphenotypes. Blood Rev..

[92] Morris C.R., Kato G.J., Poljakovic M., Wang X., Blackwelder W.C., Sachdev V., Hazen S.L., Vichinsky E.P., Morris S.M., Gladwin M.T. (2005). Dysregulated arginine metabolism, hemolysis-associated pulmonary hypertension, and mortality in sickle cell disease. JAMA.

[93] Landburg P.P., Teerlink T., Biemond B.J., Brandjes D.P., Muskiet F.A., Duits A.J., Schnog J.B., group C.s. (2010). Plasma asymmetric dimethylarginine concentrations in sickle cell disease are related to the hemolytic phenotype. Blood Cells Mol. Dis..

[94] Waugh W.H., Daeschner C.W., Files B.A., McConnell M.E., Strandjord S.E. (2001). Oral citrulline as arginine precursor may be beneficial in sickle cell disease: Early phase two results. J. Natl. Med. Assoc..

[95] Majumdar S., Tirona R., Mashegu H., Desai J., Shannon N.T., Summar M., Cunningham G., Darbari D., Nickel R., Campbell A. (2019). A phase 1 dose-finding study of intravenous L-citrulline in sickle cell disease: A potential novel therapy for sickle cell pain crises. Br. J. Haemotol..

[96] Klings E.S., Machado R.F., Barst R.J., Morris C.R., Mubarak K.K., Gordeuk V.R., Kato G.J., Ataga K.I., Gibbs J.S., Castro O. (2014). An official American Thoracic Society clinical practice guideline: Diagnosis, risk stratification, and management of pulmonary hypertension of sickle cell disease. Am. J. Respir. Crit. Care Med..

[97] Al-Ghanem G., Shah P., Thomas S., Banfield L., El Helou S., Fusch C., Mukerji A. (2017). Bronchopulmonary dysplasia and pulmonary hypertension: A meta-analysis. J. Perinatol..

[98] Mirza H., Ziegler J., Ford S., Padbury J., Tucker R., Laptook A. (2014). Pulmonary hypertension in preterm infants: Prevalence and association with bronchopulmonary dysplasia. J. Pediatr..

[99] Mourani P.M., Sontag M.K., Younoszai A., Miller J.I., Kinsella J.P., Baker C.D., Poindexter B.D., Ingram D.A., Abman S.H. (2015). Early pulmonary vascular disease in preterm infants at risk for bronchopulmonary dysplasia. Am. J. Resp. Crit. Care Med..

[100] An H.S., Bae E.J., Kim G.B., Kwon B.S., Beak J.S., Kim E.K., Kim H.S., Choi J.H., Noh C.I., Yun Y.S. (2010). Pulmonary hypertension in preterm infants with bronchopulmonary dysplasia. Korean Circ. J..

[101] Bhat R., Salas A.A., Foster C., Carlo W.A., Ambalavanan N. (2012). Prospective analysis of pulmonary hypertension in extremely low birth weight infants. Pediatrics.

[102] Slaughter J.L., Pakrashi T., Jones D.E., South A.P., Shah T.A. (2011). Echocardiographic detection of pulmonary hypertension in extremely low birth weight infants with bronchopulmonary dysplasia requiring prolonged positive pressure ventilation. J. Perinatol..

[103] Abman S.H., Accurso F.J., Bowman C.M. (1984). Unsuspected cardiopulmonary abnormalities complicating bronchopulmonary dysplasia. Arch. Dis. Child.

[104] Fouron J.C., Le Guennec J.C., Villemant D., Perreault G., Davignon A. (1980). Value of echocardiography in assessing the outcome of bronchopulmonary dysplasia of the newborn. Pediatrics.

[105] Khemani E., McElhinney D.B., Rhein L., Andrade O., Lacro R.V., Thomas K.C., Mullen M.P. (2007). Pulmonary artery hypertension in formerly premature infants with bronchopulmonary dysplasia: Clinical features and outcomes in the surfactant era. Pediatrics.

[106] Abman S.H., Wolfe R.R., Accurso F.J., Koops B.L., Bowman C.M., Wiggins J.W. (1985). Pulmonary vascular response to oxygen in infants with severe bronchopulmonary dysplasia. Pediatrics.

[107] Goodman G., Perkin R.M., Anas N.G., Sperling D.R., Hicks D.A., Rowen M. (1988). Pulmonary hypertension in infants with bronchopulmonary dysplasia. J. Pediatr..

[108] Carroll J., Rao R., Steinhorn R.H. (2024). Targeted Therapies for Neonatal Pulmonary Hypertension: Beyond Nitric Oxide. Clin. Perinatol..

[109] Abman S.H., Mullen M.P., Sleeper L.A., Austin E.D., Rosenzweig E.B., Kinsella J.P., Ivy D., Hopper R.K., Raj J.U., Fineman J. (2021). Characterisation of paediatric pulmonary hypertensive vascular disease from the PPHNet Registry. Eur. Respir. J..

[110] Fraga M.V., Dysart K.C., Stoller J.Z., Huber M., Fedec A., Mercer-Rosa L., Kirpalani H. (2023). Echocardiographic Assessment of Pulmonary Arterial Hypertension Following Inhaled Nitric Oxide in Infants with Severe Bronchopulmonary Dysplasia. Neonatology.

[111] Backes C.H., Reagan P.B., Smith C.V., Jadcherla S.R., Slaughter J.L. (2016). Sildenafil Treatment of Infants With Bronchopulmonary Dysplasia-Associated Pulmonary Hypertension. Hosp. Pediatr..

[112] Thompson E.J., Perez K., Hornik C.P., Smith P.B., Clark R.H., Laughon M., Best Pharmaceuticals for Children Act—Pediatric Trials Network Steering Committee (2019). Sildenafil Exposure in the Neonatal Intensive Care Unit. Am. J. Perinatol..

[113] van der Graaf M., Rojer L.A., Helbing W., Reiss I., Etnel J.R.G., Bartelds B. (2019). EXPRESS: Sildenafil for bronchopulmonary dysplasia and pulmonary hypertension: A meta-analysis. Pulm. Circ..

[114] Lin Y.J., Markham N.E., Balasubramaniam V., Tang J.R., Maxey A., Kinsella J.P., Abman S.H. (2005). Inhaled nitric oxide enhances distal lung growth after exposure to hyperoxia in neonatal rats. Pediatr. Res..

[115] Rudic R.D., Shesely E.G., Maeda N., Smithies O., Segal S.S., Sessa W.C. (1998). Direct evidence for the importance of endothelium-derived nitric oxide in vascular remodeling. J. Clin. Investig..

[116] Seki J., Nishio M., Kato Y., Motoyama Y., Yoshida K. (1995). FK409, a new nitric-oxide donor, suppresses smooth muscle proliferation in the rat model of balloon angioplasty. Atherosclerosis.

[117] Mourani P.M., Abman S.H. (2013). Pulmonary vascular disease in bronchopulmonary dysplasia: Pulmonary hypertension and beyond. Curr. Opin. Pediatr..

[118] Stenmark K., Abman S. (2005). Lung vascular development: Implications for the pathogenesis of bronchopulmonary dysplasia. Ann. Rev. Physiol..

[119] Alvira C.M. (2016). Aberrant Pulmonary Vascular Growth and Remodeling in Bronchopulmonary Dysplasia. Front. Med..

[120] Parker T.A., Abman S.H. (2003). The pulmonary circulation in bronchopulmonary dysplasia. Semin. Neonatol..

[121] Montgomery A.M., Bazzy-Asaad A., Asnes J.D., Bizzarro M.J., Ehrenkranz R.A., Weismann C.G. (2016). Biochemical Screening for Pulmonary Hypertension in Preterm Infants with Bronchopulmonary Dysplasia. Neonatology.

[122] Fike C.D., Aschner J.L., Avachat C., Birnbaum A.K., Sherwin C.M.T. (2023). Multi-dose enteral L-citrulline administration in premature infants at risk of developing pulmonary hypertension associated with bronchopulmonary dysplasia. J. Perinatol..

[123] Chien S.J., Lin K.M., Kuo H.C., Huang C.F., Lin Y.J., Huang L.T., Tain Y.L. (2014). Two different approaches to restore renal nitric oxide and prevent hypertension in young spontaneously hypertensive rats: L-citrulline and nitrate. Transl. Res..

[124] Persson P., Fasching A., Teerlink T., Hansell P., Palm F. (2014). L-Citrulline, but not L-arginine, prevents diabetes mellitus-induced glomerular hyperfiltration and proteinuria in rat. Hypertension.

[125] You H., Gao T., Cooper T.K., Morris S.M., Awad A.S. (2014). Diabetic nephropathy is resistant to oral L-arginine or L-citrulline supplementation. Am. J. Physiol. Ren. Physiol..

[126] Chen G.F., Baylis C. (2010). In vivo renal arginine release is impaired throughout development of chronic kidney disease. Am. J. Physiol. Ren. Physiol..

[127] Marini J.C., Didelija I.C., Fiorotto M.L. (2014). Extrarenal citrulline disposal in mice with impaired renal function. Am. J. Physiol. Ren. Physiol..

[128] Papadia C., Osowska S., Cynober L., Forbes A. (2018). Citrulline in health and disease. Review on human studies. Clin. Nutr..

[129] Summar M. (2024). Potential therapeutic uses of L-citrulline beyond genetic urea cycle disorders. J. Inherit. Metab. Dis..

